# Homologous Ad26.COV2.S vaccination results in reduced boosting of humoral responses in hybrid immunity, but elicits antibodies of similar magnitude regardless of prior infection

**DOI:** 10.1371/journal.ppat.1011772

**Published:** 2023-11-09

**Authors:** Thandeka Moyo-Gwete, Simone I. Richardson, Roanne Keeton, Tandile Hermanus, Holly Spencer, Nelia P. Manamela, Frances Ayres, Zanele Makhado, Thopisang Motlou, Marius B. Tincho, Ntombi Benede, Amkele Ngomti, Richard Baguma, Masego V. Chauke, Mathilda Mennen, Marguerite Adriaanse, Sango Skelem, Ameena Goga, Nigel Garrett, Linda-Gail Bekker, Glenda Gray, Ntobeko A. B. Ntusi, Catherine Riou, Wendy A. Burgers, Penny L. Moore

**Affiliations:** 1 SA MRC Antibody Immunity Research Unit, School of Pathology, University of the Witwatersrand, Johannesburg, South Africa; 2 Centre for HIV and STIs, National Institute for Communicable Diseases of the National Health Laboratory Services, Johannesburg, South Africa; 3 Institute of Infectious Disease and Molecular Medicine, University of Cape Town, Observatory, South Africa; 4 Division of Medical Virology, Department of Pathology; University of Cape Town; Observatory, South Africa; 5 Department of Medicine, University of Cape Town and Groote Schuur Hospital; Observatory, South Africa; 6 Cape Heart Institute, Faculty of Health Sciences, University of Cape Town; Observatory, South Africa; 7 South African Medical Research Council Extramural Unit on Intersection of Non-communicable Diseases and Infectious Diseases, University of Cape Town, Cape Town, South Africa; 8 South African Medical Research Council, Cape Town, South Africa; 9 Centre for the AIDS Programme of Research in South Africa, Durban, South Africa; 10 Discipline of Public Health Medicine, University of KwaZulu-Natal, Durban, South Africa; 11 Desmond Tutu HIV Centre, Cape Town, South Africa; 12 Wellcome Centre for Infectious Diseases Research in Africa, University of Cape Town, Observatory, South Africa; Emory University, UNITED STATES

## Abstract

The impact of previous SARS-CoV-2 infection on the durability of Ad26.COV2.S vaccine-elicited responses, and the effect of homologous boosting has not been well explored. We followed a cohort of healthcare workers for 6 months after receiving the Ad26.COV2.S vaccine and a further one month after they received an Ad26.COV2.S booster dose. We assessed longitudinal spike-specific antibody and T cell responses in individuals who had never had SARS-CoV-2 infection, compared to those who were infected with either the D614G or Beta variants prior to vaccination. Antibody and T cell responses elicited by the primary dose were durable against several variants of concern over the 6 month follow-up period, regardless of infection history. However, at 6 months after first vaccination, antibody binding, neutralization and ADCC were as much as 59-fold higher in individuals with hybrid immunity compared to those with no prior infection. Antibody cross-reactivity profiles of the previously infected groups were similar at 6 months, unlike at earlier time points, suggesting that the effect of immune imprinting diminishes by 6 months. Importantly, an Ad26.COV2.S booster dose increased the magnitude of the antibody response in individuals with no prior infection to similar levels as those with previous infection. The magnitude of spike T cell responses and proportion of T cell responders remained stable after homologous boosting, concomitant with a significant increase in long-lived early differentiated CD4 memory T cells. Thus, these data highlight that multiple antigen exposures, whether through infection and vaccination or vaccination alone, result in similar boosts after Ad26.COV2.S vaccination.

## Author summary

A single dose of the adenovirus vector-based Ad26.COV2.S vaccine has been shown to induce durable immune responses which last months after administration. However, the impact of prior infection with distinct SARS-CoV-2 variants on antibody and T cell immune response durability has not been described in detail. In this study, we assessed antibody and T cell responses elicited by Ad26.COV2.S vaccination for 6 months after the initial dose in healthcare workers who either had no prior SARS-CoV-2 infection, or who had previously been infected with either the D614G or Beta variant. Immune responses were durable for up to 6 months after a single Ad26.COV2.S vaccination. However, antibody responses were significantly higher in individuals with hybrid immunity compared to those with no prior infection. A homologous Ad26.COV2.S booster dose approximately 9 months after first vaccination increased the magnitude of the antibody responses to similar levels regardless of prior infection status. Boosting also expanded the proportion of early differentiated SARS-CoV-2 spike memory CD4 T cells. These data highlight the importance of a booster in individuals who only received a single Ad26.COV2.S dose.

## Introduction

The Ad26.COV2.S vaccine, developed by Johnson and Johnson, is an adenovirus vector-based vaccine initially rolled out as a single dose regimen that conferred 85% efficacy against severe disease caused by SARS-CoV-2 [1,2]. However, as variants of concern (VOCs) emerged, vaccine efficacy and effectiveness against infection and symptomatic disease decreased and hence, in South Africa, a booster dose was recommended [3–7]. A booster dose of the Ad26.COV2.S vaccine was reported to confer 75% protection against moderate to severe COVID-19 disease prior to the emergence of the Delta variant [8] and this was consistent with the booster vaccine effectiveness of 72% against hospitalization during the Omicron BA.1 wave in South Africa [4].

As the pandemic progressed, the proportion of those with hybrid immunity increased substantially [9]. In South Africa, it is now estimated that the majority of individuals in the population have been infected with SARS-CoV-2 [10–12]. We and others have shown that individuals infected with SARS-CoV-2 prior to, or following, vaccination have significantly increased humoral responses compared to those with infection or vaccination alone [13–16]. Specifically, Ad26.COV2.S vaccinees who were previously infected with either D614G or Beta showed significantly boosted binding, neutralizing and ADCC responses. Conversely, prior SARS-CoV-2 infection did not significantly impact the magnitude of spike-specific-CD4 or CD8 T cell responses [13]. Similar results were obtained with ChAdOx nCov-19, another adenovirus-based vaccine, where a single dose of ChAdOx nCov-19 administered to participants previously infected with SARS-CoV-2 enhanced cross-reactive neutralizing antibody responses 1 month after vaccination [17,18].

Furthermore, “immune imprinting” has been reported, where the sequence of the initial antigen exposure determined the cross-reactivity of the antibody response regardless of subsequent heterologous exposures [13,19–21]. In our previous study of Ad26.COV2.S vaccinated healthcare workers (HCWs), participants previously infected with the Beta variant had more cross-reactive antibody responses compared to those previously infected with the D614G variant [13].

Although a number of studies have now assessed the durability of immune responses to the Ad26.CoV.2S vaccine, confirming that Ad26.CoV.2S-induced antibody and T cell responses remain stable over an 8-month period, the impact of previous infection with different variants on the durability of the immune response has not been well described [22–26]. In this study, we longitudinally assessed antibody and T cell responses elicited by Ad26.COV2.S vaccination in a previously described cohort of HCWs who were either SARS-CoV-2 naïve, or had been infected with the D614G or Beta variants prior to vaccination [13]. We first defined the magnitude of spike-specific immune responses up to 6 months after vaccination and found these varied in titer, but were durable after a single Ad26.COV2.S dose, regardless of prior infection status. Secondly, we show that a homologous Ad26.COV2.S boost increased the magnitude of the antibody response to similar levels regardless of prior infection status, or the infecting variant, and expanded the proportion of early differentiated memory CD4 T cells specific for spike. Determining the durability, magnitude and boosting potential of immune responses in the context of hybrid immunity, now near universal, remains key to help inform policy on the type and frequency of booster administration, and to better understand immune protection against SARS-CoV-2.

## Results

### Antibody and T cell responses elicited by Ad26.COV2.S vaccination are maintained for up to 6 months regardless of prior infection

To determine the durability of immune responses following a single dose of Ad26.COV2.S vaccination, we examined longitudinal antibody and T cell responses in a previously-described cohort of HCWs [13]. The cohort included COVID-19 naïve participants (Group 1) and participants who had either a D614G (Group 2) or Beta (Group 3) infection prior to Ad26.COV2.S vaccination (**S1A Fig**). The median time between SARS-CoV-2 infection and vaccination was 7.2 months for Group 2 (interquartile range (IQR): 6.6–8.6) and 2.4 months for Group 3 (IQR: 1.8–2.7). Blood was drawn 22 days (IQR: 14–29) prior to vaccination for all groups (pre-vax samples) and approximately 1 month (median of 29 days, IQR: 28–34) after vaccination. A subset of these participants (n = 18, 15 and 17 for groups 1, 2 and 3, respectively) had follow-up samples taken at approximately 6 months after vaccination enabling us to perform durability studies (**S1A and S1B Fig**). Of these, 9,12 and 9 participants (groups 1, 2 and 3, respectively) received a Ad26.COV2.S booster dose approximately 9 months after the first vaccination (median of 8.8, IQR: 8.7–8.9) and blood was drawn at a median of 23 days (IQR: 21–24) after the vaccine boost (**S1A Fig**). As breakthrough infection (BTI) impacts antibody kinetics and T cell function [14,27], we screened the cohort for BTIs. Using a 2-fold increase in the nucleocapsid IgG binding values or a PCR-confirmed infection as criteria, nine individuals were identified with either Delta or Omicron breakthrough infections during the course of the study. Seven of these BTIs occurred before the 6-month time-point and two were detected at the 1-month post-boost time-point. Of note, 5/7 of participants who had a BTI prior to the 6-month time-point were from the infection-naïve Group 1.

**Fig 1:**
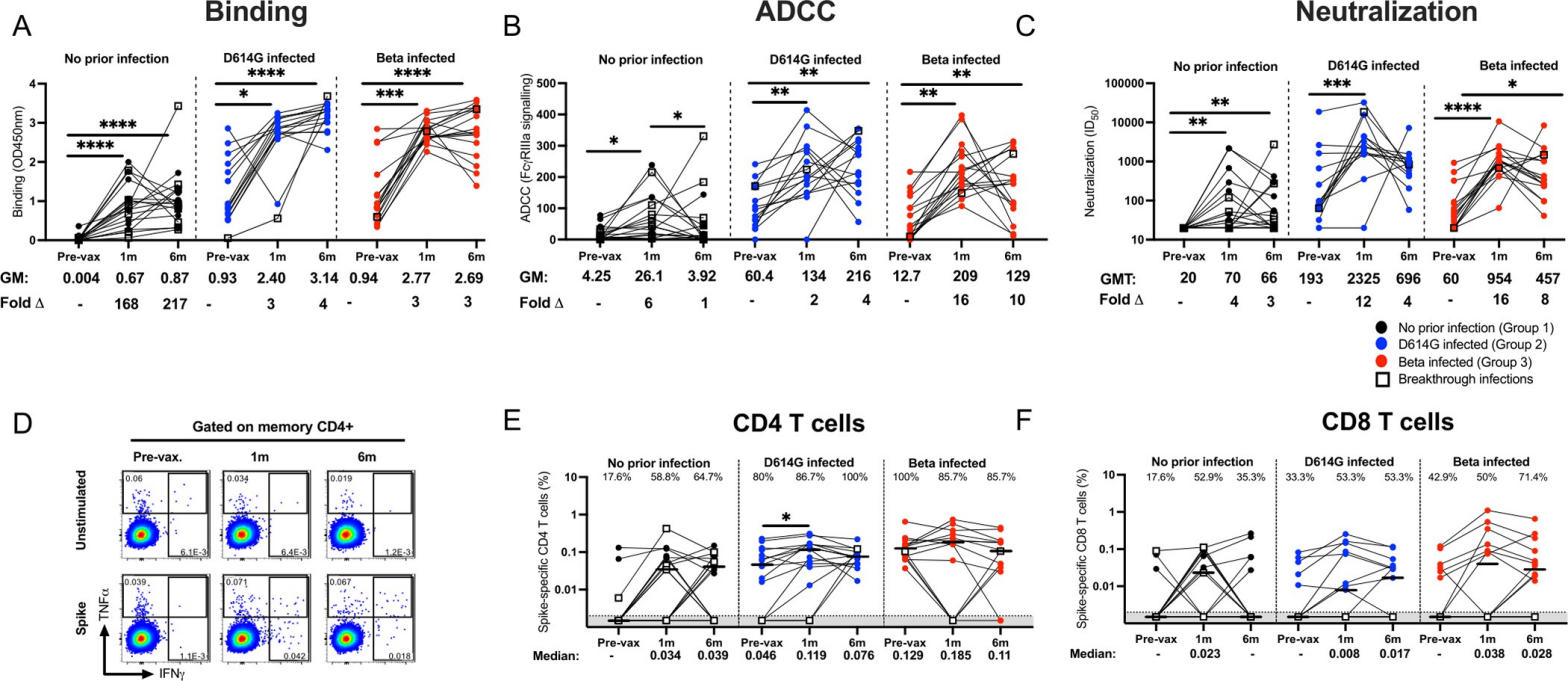
Durability of antibody and T cell responses 6 months after a single Ad26.COV2.S dose. Participants with no prior infection (black, Group 1), infected with D614G (blue, Group 2) or infected with Beta (red, Group 3) were tested for binding (**A**), antibody-dependent cellular cytotoxicity (ADCC) activity (**B**), and neutralization (**C**) of the D614G variant. Ancestral spike-specific CD4 (**D and E**) and CD8 (**F**) T cell responses before vaccination and at 1 and 6 months after vaccination. For antibody responses, geometric mean (GM) values and fold changes are shown below the graphs. Fold changes were all calculated relative to the pre-vaccination timepoints. Binding antibodies were quantified by OD450nm values, ADCC activity was measured in FcγRIIIa signaling and neutralization was indicated by 50% inhibitory dilution (ID_50_) values. All serology experiments were performed in duplicate. For T cell responses, the proportion of responders (%) is shown within the graph. Frequencies are reported after background subtraction and medians are indicated below the graph. Representative flow plots are shown in **D.** The bars in **E** and **F** represent the median response. Friedman test with Dunn’s correction for multiple comparisons was used to determine statistical significance. Significance is shown as: ****p<0.0001, ***<0.001, **p<0.01, *p<0.05.

We first longitudinally assessed the binding antibody response to D614G using a SARS-CoV-2 spike enzyme linked immunosorbent assay (ELISA), antibody dependent cellular cytotoxicity (ADCC) responses using a FcγRIIIa activation assay and neutralizing antibodies using a pseudovirus-based assay (**Fig 1A–1C**). BTIs are denoted by open symbols. As expected, antibody binding, ADCC and neutralizing responses at baseline were higher in the groups with prior infection compared to Group 1 (**S2 Fig**). Antibody binding responses against D614G significantly increased 1 month after vaccination and remained high at month 6 in all groups. As expected, geometric mean (GM) values were higher in the previously infected groups compared to Group 1 at all tested time points (**Fig 1A**).

For ADCC, infection-naïve participants in Group 1 showed a 6-fold significant increase from baseline and individuals with prior infection showed up to 16-fold increases (**Fig 1B**). While ADCC was maintained between 1 and 6 months in Groups 2 and 3 overall, there was considerable variation within the groups, with a substantial proportion of individuals either showing increased or decreased ADCC over this period. This heterogeneity was more pronounced than for the other functions we measured, likely implicating other modulators of Fc effector function including antibody isotype or subclass and glycosylation [28].

Neutralizing antibody responses against D614G were significantly increased in all three groups at 1 month after vaccination. Although reduced D614G neutralizing antibody responses were observed between 1 and 6 months post-vaccination (Group 1: geometric mean titer (GMT) of 70 vs 66; Group 2: 2,325 vs 5696 and Group 3: 954 vs 457, respectively), neutralizing activity remained detectable in most of the samples tested (**Fig 1C).** Overall, all three antibody functions were relatively durable against D614G with a slight drop in neutralizing titers and ADCC by 6 months post-vaccination. Antibody levels were significantly higher in the previously infected groups compared to SARS-CoV-2-uninfected participants at each time point tested (**Fig 1A–1C**).

Next, we assessed the durability of spike-specific T cell responses over a 6 month period by measuring IFN-γ, IL-2 and TNF-α cytokine production after stimulation with a peptide pool targeting ancestral spike (**Figs 1D and S3A–S3C**). As shown previously [13], all groups mounted robust spike-specific CD4 and CD8 T cell responses after primary vaccination (**Fig 1E and 1F**). Spike-specific CD4 T cell response frequencies were well maintained at 6 months post-vaccination in all groups (median: 0.034 vs 0.039, 0.119 vs 0.076, and 0.185 vs 0.11 for groups 1, 2 and 3 respectively) (**Fig 1E**). No appreciable change was detected in the proportion of CD4 T cell responders between 1 and 6 months after the initial vaccination in any of the tested groups. To further interrogate the quality of the SARS-CoV-2-specific response, we performed polyfunctional analysis (**S3F Fig**) to determine the proportion of triple-functional, dual-functional or mono-functional CD4 cells producing different combinations of IFN-γ, IL-2 and TNF-α. Overall, there were no significant changes in the polyfunctionality of the CD4 T cell response between the 1 month and 6 month time post vaccination (**S3F Fig pies**), although we did see a significant decrease in IFN-γ+TNF-α+ and a significant increase in IFN-γ+IL-2+ dual-producing cells at the 6 month time-point (p = 0.049 and p = 0.006 respectively; **S3F Fig**).

The magnitude of the SARS-CoV-2-specific CD8 T cell response detected at 1 month after vaccination was maintained at 6 months for groups 2 and 3. In group 1, a 17.6% reduction in the proportion of responders was detected at 6 months compared to 1 month post vaccination (35.3% vs 52.9%) with five participants losing their CD8 response. No changes were detected in the proportion of responders for group 2 but interestingly, a 21.4% increase in responders was detected at 6 months in Group 3 (50% at 1 month vs 71.4% at 6 months; **Fig 1F**). Analysis of the quality of the CD8 T cell response revealed no overall change in the polyfunctionality at 6 months compared to 1 month after vaccination (**S3G Fig**). The predominant subsets of cytokine producing CD8 T cells at all time-points were IFN-γ mono-functional and IFN-γ+TNF-α+ dual-functional cells (**S3G Fig**).

We also noted that overall, ten participants across the 3 groups gained a CD4 response and a further six gained a CD8 response at the 6 month time point. Of these, only three of the new CD4 and none of the new CD8 responders were confirmed as having had breakthrough infections (**Fig 1E and 1F**). Overall, these results demonstrate that the robust CD4 and CD8 T cell responses generated after primary vaccination are detectable 6 months later with no overall changes in polyfunctionality.

For all the three antibody functions tested, as well as CD4 and CD8 cytokine producing T cells, the comparison of participants with breakthrough infections, showed similar overall trends (**Fig 1**)

### Hybrid immunity confers high levels of cross-reactivity 6 months after vaccination, regardless of the infecting variant

**Fig 2:**
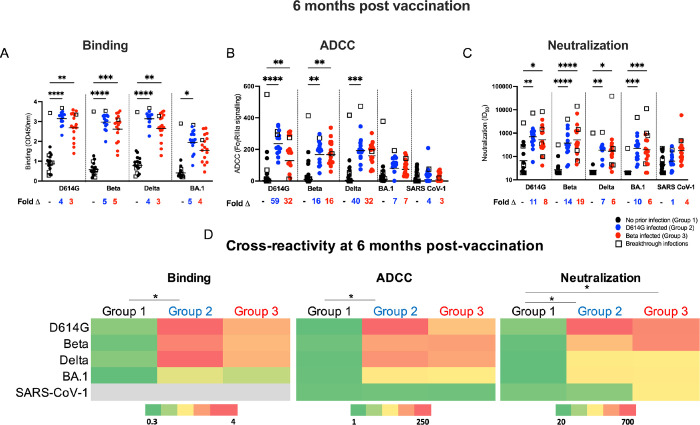
Cross-reactivity of vaccinee plasma samples at 6 months post vaccination. Plasma was tested for cross-reactivity in antibody binding (**A**), ADCC activity (**B**) and neutralization assays (**C**) against D614G, Beta, Delta, Omicron (BA.1) and SARS-CoV-1 for participants with no prior infection (black), participants infected with D614G (blue) or Beta infected participants (red). Fold changes between participant groups are shown below the graphs. Binding antibodies were quantified by OD450nm values, ADCC activity was measured in FcγRIIIa signaling and neutralization was indicated by ID_50_ values. Bars in **A** and **B** represent geometric mean values and bars in **C** represent geometric mean titers. (**D**) Heat-map depicting cross-reactivity of the plasma based on the geometric means of each group. The scale depicted shows strongest responses in red and weakest responses in green for each assay. All experiments were performed in duplicate. Statistical significance between the different groups was determined using the Kruskal-Wallis test with Dunn’s correction for multiple comparisons. Significance is shown as: ****p<0.0001, ***<0.001, **p<0.01, *p<0.05.

We next compared antibody functions against SARS-CoV-2 variants D614G, Beta, Delta and Omicron (BA.1) across the three groups 6 months after vaccination to assess the degree and durability of cross-reactivity (**Figs 2A–2C, S4, S5 and S6**). The degree of cross-reactivity for each group is summarized in **Fig 2D**, which shows the GMT against D614G, Beta, Delta and Omicron variants and SARS-CoV-1 for binding antibodies, neutralization and ADCC. Although the overall dynamics of antibody responses mirrored those observed for D614G, titers against the VOCs and SARS-CoV-1 were much lower as expected **(S4, S5 and S6 Figs)**. Binding responses were significantly higher in the previously infected Groups 2 and 3, compared to Group 1, with up to 5-fold differences in binding potency (**Fig 2A**). This is likely an underestimate of these fold changes, as binding antibody values in Groups 2 and 3 reached the upper limit of detection of the assay. The previously infected groups also displayed substantially elevated ADCC and neutralization activity towards SARS-CoV-2 variants compared to Group 1 (up to 33-fold and 19-fold, respectively) (**Fig 2B and 2C**). Interestingly, despite differences in the infecting variants and the time between infection and vaccination (7 and 2 months, respectively) before vaccination, Group 2 and Group 3 had similar levels of all three antibody functions (**Fig 2A–2D**) except for titers against SARS-CoV-1, which trended higher for Group 3 compared to Group 2 (**Fig 2C and 2D**). Thus, while early immune imprinting resulted in slightly different patterns of cross-reactivity at 1 month post-vaccination [13], these differences did not persist at 6 months after vaccination in this cohort.

### Prior infection dampens the boosting of antibody responses

**Fig 3:**
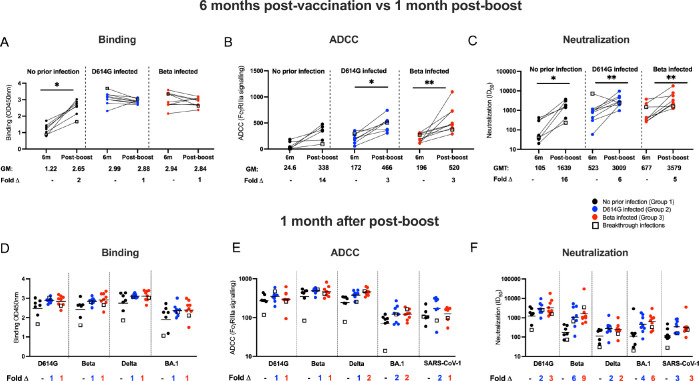
The antibody response after an Ad26.COV2.S booster dose. Antibody responses were measured pre-boost at 6 months after primary dose vaccination and compared to responses at one month after a homologous boost. Plasma samples from patients with either no prior infection (black), infected with D614G (blue), and infected with Beta (red) were tested for binding (**A**), antibody-dependent cellular cytotoxicity (ADCC) activity (**B**) and neutralization (**C**) to the D614G variant. Geometric mean values are shown below the graphs. Binding antibodies were quantified by OD450nm values, ADCC activity was measured in FcγRIIIa signaling and neutralization was indicated by ID_50_ values. All experiments were performed in duplicate. The Wilcoxon matched-pairs signed rank test was used to determine statistical significance between the 6 months post-vaccination and one month post-boost time points. Significance is shown as: **p<0.01, *p<0.05. Binding (**D**), ADCC activity (**E**) and neutralizing (**F**) responses between groups were tested one month after the booster dose. Bars in **D** and **E** represent geometric mean and bars in **F** represent geometric mean titer. Plasma activity was tested against the D614G, Beta, Delta and BA.1 variants and SARS-CoV-1. Fold changes are shown below the graphs. All experiments were performed in duplicate. Statistical significance between the different groups was determined using the Kruskal-Wallis test with Dunn’s correction for multiple comparisons. None of the comparisons were statistically significant.

Approximately 9 months after the initial Ad26.COV2.S dose, a subset of participants received an additional Ad26.COV2.S vaccine (n = 9, 12 and 9 in Group 1, 2 and 3, respectively, **S1A Fig**). Antibody responses against the D614G variant measured 6 months after the first dose were compared to those measured one month after the boost (**Fig 3**). In the SARS-CoV-2 naïve Group 1 participants, boosting with the Ad26.COV2.S vaccine resulted in a 2-fold increases in binding activity (**Fig 3A**), 14-fold increases in ADCC activity (**Fig 3B**) and 16-fold increases in neutralization titers (**Fig 3C**). The fold increase was lower in participants with prior infection, with no change in binding antibodies, a 3-fold increase in ADCC in groups 2 and 3 and a 5–6 fold increase in neutralizing activity in participants with prior infection after a Ad26.COV2.S booster (**Fig 3B and 3C**). Importantly, one month after the vaccine boost, the antibody binding titer and ADCC activity levels were comparable across all groups, irrespective of the SARS-CoV-2 variant tested (**Fig 3D and 3E)**. Although not statistically significant, neutralization potency against the Beta and BA.1 variants was higher in Groups 2 and 3 compared to Group 1 (6- and 9-fold for Beta and 4- to 6-fold for BA.1, respectively) (**Fig 3F**).

### Ad26.COV2.S boosting increases the proportion of SARS-CoV-2-spike-specific early differentiated memory T cells

**Fig 4:**
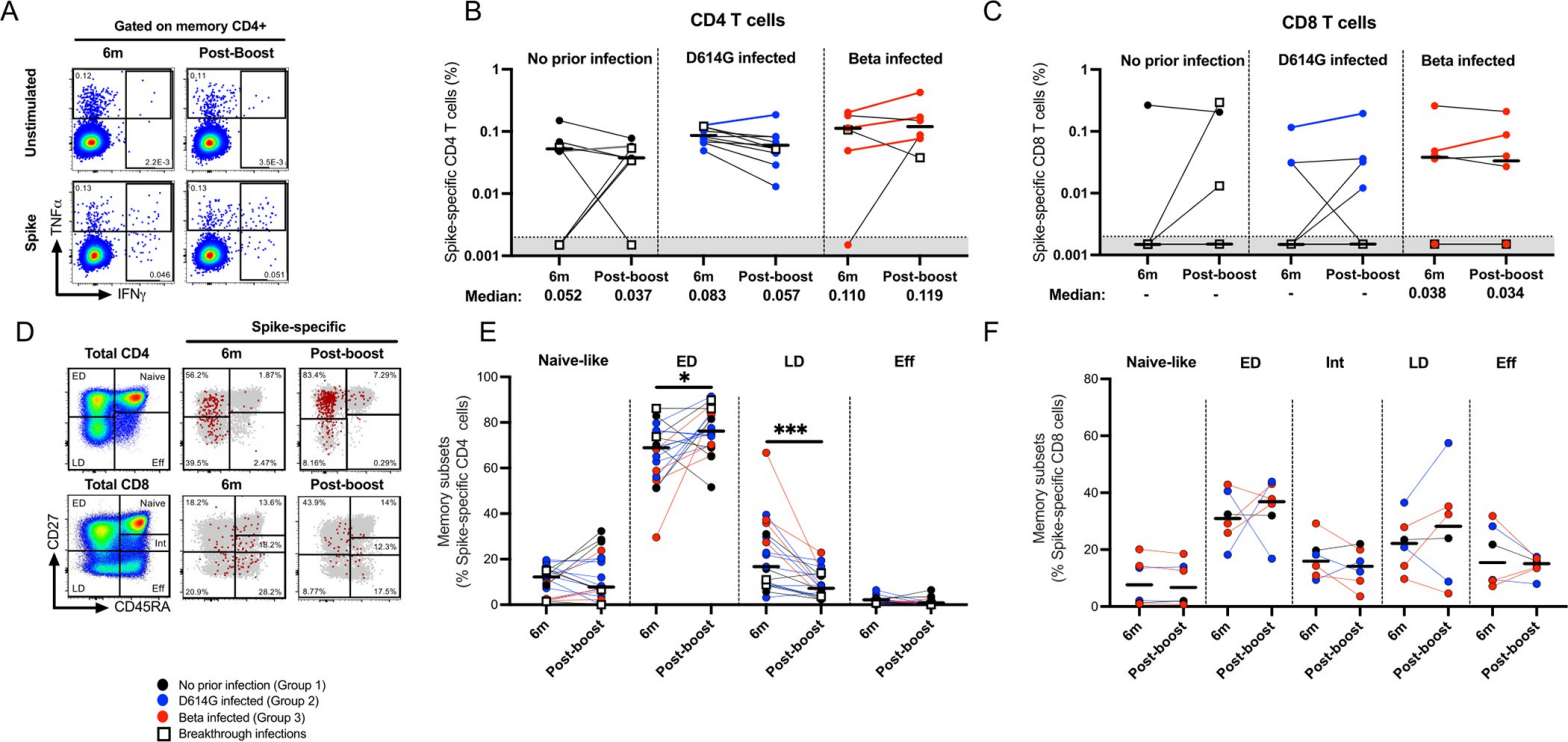
T cell response and phenotyping after an Ad26.COV2.S booster dose. T cell cytokine responses were measured at 6 months post-vaccination and compared to responses at one month after a homologous boost. PBMCs from patients with either no prior infection (black), infected with D614G (blue), or infected with Beta (red) were tested for frequency of spike-specific CD4+ (**A and B**) and CD8+ T cells (**C**) producing any of the studied cytokines (IFN-γ, IL-2 and TNF-α) in response to ancestral peptide pools. Bars represent medians with values indicated below the graphs and the colored lines represent participants with increased cytokine responses. Data are plotted as background subtracted total cytokine responses. Due to high TNF-α background, single TNF-α producing cells were excluded from analysis. Representative plots are shown in **A**. Representative overlay flow plots of CD27 and CD45RA expression are shown in **D**. Red dots depict SARS-CoV-2-spike-specific expression at 6 months (middle) and post-boost (right). Grey dots represent total CD4 (upper panel) or CD8 T cells (lower panel). Using CD27 and CD45RA, four memory subsets can be delineated (left panel): naive (CD27+CD45RA+), early differentiated (ED; CD27+CD45RA-), late differentiated (LD; CD27-CD45RA-) and effector (Eff; CD27-CD45RA+) subsets. Summary graphs of the frequency (%) of each subset within SARS-CoV-2-spike-specific CD4 (**E**) or CD8 T cells (**F**). Wilcoxon matched-pairs signed rank test was used to determine statistical significance. Significance is shown as: ***<0.001, **p<0.01, *p<0.05.

To determine the effect of a homologous vaccine booster on T cell immunity, we compared the magnitude, quality and memory differentiation phenotype of CD4 and CD8 T cells 6 months after the primary Ad26.COV2.S vaccine and 1 month after the boost in participants for which PBMC samples were available at both time points (n = 8, 10 and 6 for groups 1, 2, and 3, respectively) (**Fig 4A–4C**). No significant changes were detected in the magnitude of the spike-specific T cell response or the proportion of responders for either CD4 or CD8 T cells before and after boosting in any of the groups. There was however some heterogeneity at the individual level after boosting, with 5/24 participants across all groups having CD4 T cell responses demonstrating increased magnitudes indicated by a fold-change greater that 1.2 (**Fig 4B**) and four gaining a response. Similarly, CD8 T cells had higher cytokine responses in 2/24 participants and four gained a response (**Fig 4C**). The vaccine boost did not induce any overall change in the polyfunctional profile of either CD4 or CD8 cells (**S3F and S3G Fig**) but an increase in the proportion of dual-functional IFN-γ+TNF-α+ as well as monofunctional IFN-γ and TNF-α CD4 T cells was detected at the post-boost time-point (**S3F Fig**).

To investigate the phenotypic characteristics of spike-specific T cell responses, we analyzed their memory differentiation profiles (**Figs 4D–4F, S3A, S3D and S3E**). The measurement of CD45RA and CD27 enabled the detection of four distinct CD4 T cell populations, namely naïve-like (CD45RA+CD27+), early differentiated (ED: CD45RA-CD27+), late differentiated (LD: CD45RA-CD27-) and effector (Eff: CD45RA+CD27-) populations, with CD8 T cells having a fifth population of intermediate cells (Int: CD45RA+CD27low) (**Fig 4D**) [29–34]. Due to the low number of responders meeting the threshold for phenotypic analysis, we did a bulk analysis combining the three groups. The memory profile of the spike-specific CD4 T cells after the booster vaccination displayed a significant increase in ED (p = 0.032) and a concomitant decrease in LD (p < 0.0001) subsets compared to the 6-month time-point (median: 68.2% vs 74.9% and 21.9% vs 7.2% respectively; **Fig 4E**). No significant differences were detected among the CD8 T cell memory populations (**Fig 4F**). It should be noted that the responses of participants with a prior breakthrough infection did not cluster separately from those without prior breakthrough infection either for magnitude, functionality or phenotype.

## Discussion

Although a single dose of the Ad26.COV2.S vaccine has been shown to induce durable adaptive immune responses [22,25], the effect of previous infection on the titer and longevity of these responses and the effect of a homologous boost have not been fully explored. Our data show that while antibody activity elicited by a single Ad26.COV2.S dose was durable up to 6 months after initial vaccination, Fc functionality, antibody neutralization and binding titers remained significantly lower in individuals with no prior infection compared to individuals with hybrid immunity. The significance of this is highlighted by the fact that of the seven breakthrough infections over this time-period, most (5/7) were in individuals without hybrid immunity. A homologous vaccine booster resulted in all antibody responses reaching a similar magnitude irrespective of prior SARS-CoV-2 infection, however the magnitude of boosting in the context of hybrid immunity was reduced compared to that of previously uninfected participants. Both CD4 and CD8 SARS-CoV-2 spike-specific T cell responses were sustained 6 months after vaccination in all groups. However, unlike the antibody responses, a homologous boost did not increase the magnitude or proportion of T cell responders but did result in an increase in the proportion of SARS-CoV-2-specific early differentiated CD4 T cell memory subset.

This cohort of healthcare workers was intensively monitored for breakthrough infections throughout the study period. Although the sample size is relatively small, especially in the post-boost analyses, these highly curated data are valuable in understanding the adaptive immune response after homologous Ad26.COV2.S boosting in individuals with and without prior infection. These data suggest that boosting with the Ad26.COV2.S vaccine is beneficial for increasing antibody titers in individuals with no history of prior infection and inducing long-lived CD4 memory T cells in all groups. A smaller subset (due to loss in follow-up) was studied to compare responses between the two groups post-boost. At the pre-boost time point (6 months after initial vaccination), the differences between the prior infection and the hybrid immune groups were lower than for the larger subset that was studied longitudinally. However, the binding responses were 2–3 fold higher, the ADCC activity was 7–8 fold higher and the neutralization activity was 5–6 fold higher in the hybrid immune groups as compared to those with no prior infection. A larger sample size would be needed to further validate these findings.

We and others have shown that breakthrough infection after vaccination yields substantially higher neutralization, binding and Fc effector functions compared to vaccination alone [14,27,35] and leads to better protection from hospitalization and severe disease [36]. Here, we extend our previous observation that infection prior to Ad26.COV2.S vaccination results in superior responses compared to vaccination alone. This latter form of hybrid immunity is likely the most relevant current immune scenario for South Africa, which has extremely high population seropositivity [10–12]. Although baseline values between the three groups differ, especially between the group with no prior infection before vaccination and the groups who either had D614G or Beta infections prior to vaccination, each group is compared to their own baseline samples.

Interestingly, our study differs from the ChAdOx1 nCoV-19 vaccine where individuals who were seropositive at the start of the study did not have increased neutralizing titers after the second dose of the vaccine [18,37]. The ChAdOx1 nCoV-19 vaccine, also an adenovirus-based vaccine but not based on the stabilized spike protein, elicited antibodies that were maintained for up to 6 months post-vaccination in seropositive individuals, but large drops were observed in seronegative individuals [18]. In our study, both baseline seronegative and seropositive groups maintained their antibody levels. This may be a consequence of the stabilized spike in combination with the adenoviral delivery system used in the Ad26.COV2.S vaccine inducing a more durable response, differences in infection timing or participant characteristics between the studies.

We found that in individuals with prior infection, ADCC is only slightly boosted after a single Ad26.COV2.S dose. Additionally, ADCC shows more heterogeneity in decay of activity compared to neutralization over 6 months in vaccinees with prior infection. Others have found that the Fc response is not only quantitatively higher for individuals who received mRNA vaccination with prior infection but also qualitatively superior [38]. While known to occur following Ad26.COV2.S administration, Fc effector function has not previously been assessed longitudinally or in the context of Ad26.COV2.S hybrid immunity [39,40]. This study focused on binding, neutralization and ADCC, but there are several additional antibody functions that could be examined in the context of hybrid immunity, including antibody dependent cellular phagocytosis and complement deposition. Therefore, assessing whether our findings are applicable beyond ADCC for adenoviral vectors would be beneficial.

Different SARS-CoV-2 variants have been shown to elicit varying immune responses, which may also impact the quantity and quality of the subsequent vaccine-induced responses [13,41–43]. This phenomenon, known as “immune imprinting”, has been widely reported for influenza infection and vaccination [44,45]. Previously, we reported that prior infection one month after Ad26.COV2.S vaccine primes the immune system in different ways, depending on the infecting variant [13]. In this study, we saw slightly higher neutralization of Beta and BA.1 in Group 2 (infected with Beta) one month after a homologous boost. However, overall, we did not find strong evidence for immune imprinting leading to differing cross-reactivity at 6 months after vaccination in individuals who were infected with the D614G or Beta variants prior to vaccination. This may be a consequence of antibody maturation by somatic hypermutation leading to broadening of responses regardless of the prior infecting variant. There is evidence of continual somatic hypermutation as a result of persistent viral antigen in the gut after SARS-CoV-2 infection as well as persistence of germinal centers for up to 3 months after BNT162b2 vaccine [46–48].

Analysis of the durability of the SARS-CoV-2 specific T cell response highlights that hybrid immunity consisting of D614G or Beta infection in combination with Ad26.COV2.S vaccination does not confer an advantage with respect to T cell responses over a 6 month period as demonstrated by the similar magnitude and quality of the T cell response at 6 months compared to 1 month after vaccination. This is supported by a similar study which demonstrated equivalent magnitude and durability of T cell responses after mRNA vaccination in individuals with or without prior infection [49]. Ad26.COV2.S boosting also did not significantly increase the T cell response in the three groups tested, in agreement with the findings of others [24,50]. This is in keeping with our recent findings demonstrating that increasing numbers of SARS-CoV-2 exposures, whether through vaccinations or infections, do not result in sequential increases in the magnitude of the spike-specific T cell response [51]. Emerging data on the SARS-CoV-2 spike-specific T cell immune responses to different booster vaccination strategies suggest that heterologous boosting may offer an advantage over homologous boosting, with numerous studies now showing an increased T cell response when an mRNA boost is administered after the Ad26.COV2.S primary vaccine [24,50,52].

Despite the lack of an enhanced T cell cytokine response, either in magnitude or polyfunctionality, stimulated by Ad26.COV2.S booster vaccination, we did observe a significant increase in SARS-CoV-2-specific early differentiated memory T cells (ED: CD27+,CD45RA-). An earlier study demonstrated that after a primary mRNA vaccination regimen, effector memory 1 (EM1) T cells (CCR7+,CD27+,CD45RA-), which form a subset of ED cells, were maintained over a 6 month period [49]. Notably, they found that a high proportion of EM1 T cells in the peak CD4 T cell response was associated with increased durability of the overall T cell response. In that study, the inclusion of CCR7 enabled the delineation of early differentiated memory T cells described in our study (CD27+,CD45RA-) into central memory (CCR7-,CD27+,CD45RA-) and EM1 (CCR7+,CD27+,CD45RA-) cells. Goel et al [49] highlight the contribution of EM1 T cells to the long-lived memory CD4 T cell response, supporting the possibility that the increased early differentiated memory T cell population (of which EM1 are a subset) observed in our study after booster vaccination may result in an increase in the durability of the CD4 T cell response in these boosted participants.

Altogether, in the context of countries such as South Africa where, either through infection or vaccination, SARS-CoV-2 seropositivity is extremely high in some areas [10], a booster vaccination for individuals who received one dose of Ad26.COV2.S is likely to still be beneficial to the humoral and cellular response, particularly in the context of the high proportion of breakthrough infections that occurred in the no prior infection group.

## Methods

### Ethics statement

The study was approved by the University of Cape Town Human Research Ethics Committee (Ethics number: HREC 190/2020 and 209/2020) and the University of the Witwatersrand Human Research Medical Ethics Committee (Ethics number: M210429). Written informed consent was obtained from all participants.

### Cohort description

Healthcare workers (HCWs; n = 400) were enrolled in a longitudinal study from Groote Schuur Hospital (Cape Town, Western Cape, South Africa) as described previously [13]. Here, we investigated fifty participants who fell into one of three groups: (i) No evidence of previous SARS-CoV-2 infection by diagnostic PCR test or serial serology; (ii) infection during the ‘first wave’ of the pandemic in South Africa, prior to 1 September 2020, with known date of PCR-confirmed SARS-CoV-2 infection; and (iii) infection during the ‘second wave’, with known date of PCR-confirmed SARS-CoV-2 infection between 1 November 2020 and 31 January 2021. These 50 participants were part of a subset of 60 participants (n = 20 for each group) from an earlier study [13].

### SARS-CoV-2 spike IgG and nucleocapsid enzyme linked immunosorbent assay

SARS-CoV-2 D614G, Beta, Delta and Omicron (BA.1) variant spike proteins were expressed in Human Embryonic Kidney (HEK) 293F suspension cells by transfecting the cells with the spike plasmid. After incubating for six days at 37°C, 70% humidity and 10% CO_2_, proteins were first purified using a nickel resin followed by size-exclusion chromatography. Relevant fractions were collected and frozen at -80°C until use. Two μg/mL of spike or nucleocapsid (BioTech Africa; Catalogue number: BA25-P) protein were used to coat 96-well, high-binding plates and incubated overnight at 4°C. The plates were incubated in a blocking buffer made up of 1x PBS, 5% skimmed milk powder, 0.05% Tween 20. Plasma samples were diluted to 1:100 in a blocking buffer and added to the plates. Secondary antibody was diluted to 1:3000 in blocking buffer and added to the plates followed by TMB substrate (Thermo Fisher Scientific). Upon stopping the reaction with 1 M H_2_SO_4_, absorbance was measured at a 450nm wavelength. Antibodies CR3022, 946-E4, BD23, P2B-2F6 and 084-7D were used as positive controls and to differentiate between variants. The human anti-nucleocapsid 1A6 antibody (Thermo Fisher Scientific; Catalogue number: MA535941) was used as a positive control for the nucleocapsid ELISA. Palivizumab was used as a negative control in all experiments.

### Pseudovirus neutralization assay

SARS-CoV-2 pseudotyped lentiviruses were prepared by co-transfecting the HEK293T cell line with either SARS-CoV-2 D614G, Beta (L18F, D80A, D215G, K417N, E484K, N501Y, D614G, A701V, Δ242–244), Delta (T19R, R158G L452R, T478K, D614G, P681R, D950N, 156–157 del) or BA.1 (Omicron) (A67V, Δ69–70, T95I, G142D, Δ143–145, Δ211, L212I, 214EPE, G339D, S371L, S373P, S375F, K417N, N440K, G446S, S477N, T478K, E484A, Q493R, G496S, Q498R, N501Y, Y505H, T547K, D614G, H655Y, N679K, P681H, N764K, D796Y, N856K, Q954H, N969K, L981F) spike plasmids in conjunction with a firefly luciferase encoding lenti- virus backbone plasmid. For the neutralization assay, heat-inactivated plasma samples from vaccine recipients were incubated with the SARS-CoV-2 pseudotyped virus for 1 h at 37°C, 5% CO_2_. Subsequently, 1x10^4^ HEK293T cells engineered to overexpress ACE-2 were added and incubated at 37°C, 5% CO_2_ for 72 h upon which the luminescence of the luciferase gene was measured. CB6, CA1 and 084-7D were used as positive controls and to differentiate between variants.

### FcγRIIIa activation (Antibody dependent cellular cytotoxicity) assay

The ability of plasma antibodies to cross-link *FcγRIIIa* and spike expressing cells was measured as a proxy for antibody dependent cellular cytotoxicity (ADCC). HEK293T cells were transfected with 5 μg of SARS-CoV-2 original variant spike (D614G), Beta, Delta or Omicron (BA.1) spike plasmids and incubated for 2 days at 37°C. Expression of spike was confirmed by binding of antibodies CR3022, P2B-2F6 and AIRU946-A6 and their detection by anti-IgG APC staining measured by flow cytometry. Subsequently, 1x10^5^ spike transfected cells per well were incubated with heat inactivated plasma (1:100 final dilution) or control mAbs (final concentration of 100 μg/mL) in RPMI 1640 media supplemented with 10% FBS 1% Pen/Strep (GIBCO, Gaithersburg, MD) for 1 h at 37°C. Jurkat-Lucia NFAT-CD16 cells were added and incubated for 24 h at 37°C, 5% CO_2_. Twenty μL of supernatant was then transferred to a white 96-well plate with 50 μL of reconstituted QUANTI-Luc secreted luciferase and read immediately. Relative light units (RLU) of a no antibody control were subtracted as background. Palivizumab was used as a negative control, CR3022 and AIRU946-A6 as positive controls and P2B-2Fb to differentiate variants. To induce the transgene, 1x cell stimulation cocktail (Thermo Fisher Scientific) and 2 μg/mL ionomycin in R10 was added as a positive control. RLUs for D614G, Beta, Delta and BA.1 variants were normalized using CR3022 and AIRU946-A6, which bind similarly to the variants.

### Isolation of PBMC

Blood from heparin tubes was processed within 3 h of collection. Peripheral blood mononuclear cells (PBMC) were isolated using Ficoll-Paque by density gradient sedimentation (Amersham Biosciences, Little Chalfont, UK) as per the manufacturer’s instructions and cryopreserved in freezing media consisting of heat-inactivated fetal bovine serum (FBS, Thermo Fisher Scientific) containing 10% DMSO and stored in liquid nitrogen until use.

### Cell stimulation and flow cytometry staining

Cryopreserved PBMC were thawed, washed and rested in RPMI 1640 containing 10% heat-inactivated FCS for 4 h, seeded in a 96-well V-bottom plate at ∼2 × 10^6^ PBMC per well and stimulated with SARS-CoV-2 spike peptide pools at a final concentration of 1 μg/mL (PepTivator, Miltenyi Biotech, Bergisch Gladbach, Germany). These peptides were a combination of two commercially available peptide pools (PepTivator, Miltenyi Biotech, Bergisch Gladbach, Germany) and are 15-mer sequences with 11 amino acids (aa) overlap based on the Wuhan-1 strain. They cover the N-terminal S1 domain of SARS-CoV-2 from aa 1 to 692, as well as the majority of the C-terminal S2 domain. All stimulations were performed in the presence of Brefeldin A (10 μg/mL, Sigma-Aldrich, St Louis, MO, USA) and co-stimulatory antibodies against CD28 (clone 28.2) and CD49d (clone L25) (1 μg/mL each; BD Biosciences, San Jose, CA, USA). As a negative control, PBMC were incubated with co-stimulatory antibodies, Brefeldin A and an equimolar amount of DMSO.

Cells were stimulated for 16 h, washed and stained with LIVE/DEAD Fixable VIVID Stain (Invitrogen, Carlsbad, CA, USA) prior to surface staining with the following antibodies: Pac Blue (TuK4, Invitrogen Thermo Fisher Scientific), CD19 Pac Blue (SJ25-C1, Invitrogen Thermo Fisher Scientific), CD4 PerCP-Cy5.5 (L200, BD Biosciences, San Jose, CA, USA), CD8 BV510 (RPA-8, Biolegend, San Diego, CA, USA), CD27 PE-Cy5 (1A4, Beckman Coulter), CD45RA BV570 (HI100, Biolegend, San Diego, CA, USA). Cells were then fixed and permeabilized using a Cytofix/Cyto perm buffer (BD Biosciences) and stained with CD3 BV650 (OKT3) IFN-γ Alexa 700 (B27), TNF-α BV786 (Mab11) and IL-2 APC (MQ1-17H12) from Biolegend. Finally, cells were washed and fixed in CellFIX (BD Biosciences). Samples were acquired on a BD LSR-II flow cytometer and analyzed using FlowJo (v10, FlowJo LLC, Ashland, OR, USA). Analysis of cytokine co-expressing populations was performed using Pestle and SPICE version 5.1. A median of 272 380 CD4 events (IQR: 216 796–355 414) and 147 695 CD8 events (IQR: 109 697–202 204) were acquired. For cytokine analysis, cells were gated on singlets, CD14- CD19-, live lymphocytes, CD4 and CD8 and memory cells (excluding naive CD27+ CD45RA+ population). Results are expressed as the frequency of CD4+ or CD8+ memory T cells expressing IFN-γ, TNF-α or IL-2. For memory phenotyping, cells were gated on singlets, CD14- CD19-, live lymphocytes, CD4 and CD8 followed by IFN-γ and IL-2 with memory cells being gated on the population of SARS-CoV-2-specific CD4+ and CD8+ T cells producing IFN-γ and/or IL-2 subset. Using CD27 and CD45RA, four memory subsets can be delineated: naive (CD27+CD45RA+), early differentiated (ED; CD27+CD45RA-), late differentiated (LD; CD27-CD45RA-) and effector (Eff; CD27-CD45RA+) subsets [29–34]. To ensure the robustness of phenotyping, we applied a cut-off of 40 SARS-CoV-2 specific T cell cytokine events. Due to high TNF-α background levels, cells producing TNF-α alone were excluded from the analysis.

### Statistical analysis

The Friedman test with Dunn’s correction for multiple comparisons was used to determine statistical significance in paired samples. Significance is shown as: ****p<0.0001, ***<0.001, **p<0.01, *p<0.05. Statistical significance between the geometric mean values of the three groups shown in the heat map as well as between the antibody baseline values for each group was determined using the Kruskal-Wallis test with Dunn’s correction for multiple comparisons. Heat maps were generated using Microsoft Excel. Significance is shown as: ****p<0.0001, ***<0.001, **p<0.01, *p<0.05. The Wilcoxon matched-pairs signed rank test was used to determine statistical significance between the 6 months post-vaccination and one month post-boost time points. Significance is shown as: **p<0.01, *p<0.05. Wilcoxon matched-pairs signed rank test was also used to determine statistical significance for the T cell assays. Significance is shown as: ***<0.001, **p<0.01, *p<0.05. Geometric mean or median values and fold changes are shown under the relevant graphs. Analyses were conducted using Graphpad Prism v10.0.2.

## Supporting information

S1 Fig Study design and participant demographics. (**A and B**) Schematic representing the study design and participant demographics. Participants were categorized into three groups: Group 1: no infection prior to vaccination (n = 13), Group 2: D614G-infected prior to vaccination (n = 14) and Group 3: Beta-infected prior to vaccination (n = 16). All three groups were followed for 6 months after initial vaccination. A subset of n = 6 Group 1, n = 10 Group 2 and n = 8 Group 3 participants received a homologous Ad26.COV2.S vaccination and boosting effect was analyzed after 1 month. Schematic was created using BioRender.com.
(TIFF)

S2 Fig Comparison of antibody baseline values between the three groups. Plasma was tested at baseline (pre-vax) for antibody binding (**A**), ADCC activity (**B**) and neutralization assays (**C**) against D614G, Beta, Delta, Omicron (BA.1) and SARS-CoV-1 for participants with no prior infection (black), participants infected with D614G (blue) or Beta infected participants (red). Responses were compared between the three groups. Binding antibodies were quantified by OD450nm values, ADCC activity was measured in FcγRIIIa signaling and neutralization was indicated by ID50 values. Bars for binding and ADCC represent geometric mean values and bars for neutralization represent geometric mean titer. Statistical significance between the different groups was determined using the Kruskal-Wallis test with Dunn’s correction for multiple comparisons. Significance is shown as: ****p<0.0001, ***<0.001, **p<0.01, *p<0.05.
(TIFF)

S3 Fig Flow cytometry gating strategy. Gating strategy **(A and B)** and representative examples of SARS-CoV-2 spike-specific IFN-γ, IL-2 and TNF-α production in memory CD4+ and CD8+ T cells (**C**). (**D**) Representative examples of SARS-CoV-2 spike-specific IFN-γ or IL-2 production in CD4+ and CD8+ T cells on which memory phenotyping were gated. (**E**) Representative plots showing spike-specific T cell immune phenotyping. ED: early differentiated, LD: late differentiated, Eff: effector, Inter: intermediate. Polyfunctional profile of SARS-CoV-2-specific CD4+ (**F**) and CD8+ T cells (**G**) at the 1M (red bar), 6M (green bar) and post boost (blue bar) time-points. PBMCs from patients with either no prior infection (black), infected with D614G (blue), or infected with Beta (red) are represented by closed circles and BTIs are represented as white squares. The x-axis illustrates each combination which is indicated with a black circle for the presence of IFN-g, IL-2 and TNF-a. The medians and interquartile range are shown. Each response pattern (any possible combination of IFN-g, IL-2 and TNF-a production) is color coded and summarized in the pie charts, with each pie slice representing the median contribution of each combination to the total SARS-CoV-2 responses. The permutation test was used to compare the statistical differences between the pie charts and the Mann Whitney Sum Test to compare response patterns between the time-points; p values <0.05 were considered statistically significant and are bolded.
(TIFF)

S4 Fig Durability of binding responses 6 months after a single dose of Ad26.COV2.S. Plasma samples from participants with no prior infection (black), infected with D614G (blue) or infected with Beta (red) were tested for binding responses to the Beta (**A**), Delta (**B**) and BA.1 (**C**) variants at three time points (pre-vaccination, 1 and 6 months post-vaccination). Geometric mean values and median fold changes are shown below the graphs. Fold changes were all calculated relative to the pre-vaccination time points. Binding antibodies were quantified by OD450nm values. All experiments were performed in duplicate. The Friedman test with Dunn’s correction for multiple comparisons was used to determine statistical significance. Significance is shown as: ****p<0.0001, ***<0.001, **p<0.01, *p<0.05.
(TIFF)

S5 Fig Durability of antibody dependent cellular cytotoxicity 6 months after a single dose of Ad26.COV2.S. Plasma samples from participants with no prior infection (black), infected with D614G (blue) or infected with Beta (red) were tested for ADCC activity to the Beta (**A**), Delta (**B**), BA.1 (**C**) variants and SARS-CoV-1 (**D**) at three time points (i.e, pre-vaccination, 1 and 6 months post-vaccination). Geometric mean values and median fold changes are shown below the graphs. Fold changes were all calculated relative to the pre-vaccination timepoints. ADCC activity was measured in FcγRIIIa signaling. All experiments were performed in duplicate. The Friedman test with Dunn’s correction for multiple comparisons was used to determine statistical significance. Significance is shown as: ****p<0.0001, ***<0.001, **p<0.01, *p<0.05.
(TIFF)

S6 Fig Durability of neutralizing responses 6 months after single Ad26.COV2.S dose. Plasma samples from participants with either no prior infection (black), infected with D614G (blue), and infected with Beta (red) were tested for neutralizing responses to the Beta (**A**), Delta (**B**), BA.1 (**C**) variants and SARS-CoV-1 (**D**). Plasma was tested over three time points; pre-vaccination, 1 and 6 months post-vaccination. Geometric mean values and fold changes are shown below the graphs. Fold changes were all calculated relative to the pre-vaccination timepoints. Neutralization was indicated by ID_50_ values. All experiments were performed in duplicate. The Friedman test with Dunn’s correction for multiple comparisons was used to determine statistical significance. Significance is shown as: ****p<0.0001, ***<0.001, **p<0.01, *p<0.05.
(TIFF)
