# Supplementary figures and images for: Homologous Ad26.COV2.S vaccination results in reduced boosting of humoral responses in hybrid immunity, but elicits antibodies of similar magnitude regardless of prior infection
Source: PLoS Pathog. 2023 Nov 9;19(11):e1011772. doi: 10.1371/journal.ppat.1011772 (PMC10684107; doi:10.1371/journal.ppat.1011772)

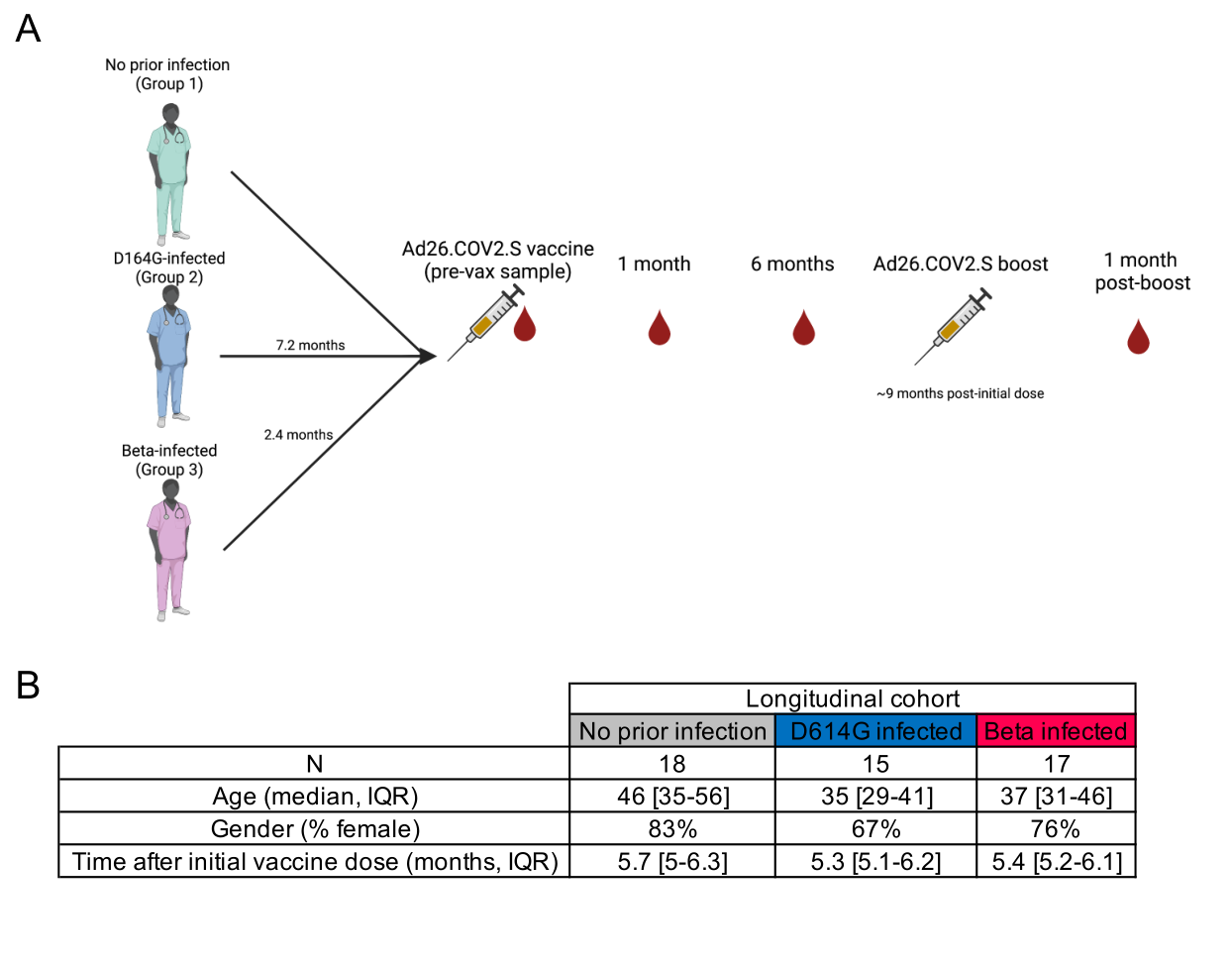

Supplement: S1 Fig — (A and B) Schematic representing the study design and participant demographics. Participants were categorized into three groups: Group 1: no infection prior to vaccination (n = 13), Group 2: D614G-infected prior to vaccination (n = 14) and Group 3: Beta-infected prior to vaccination (n = 16). All three groups were followed for 6 months after initial vaccination. A subset of n = 6 Group 1, n = 10 Group 2 and n = 8 Group 3 participants received a homologous Ad26.COV2.S vaccination and boosting effect was analyzed after 1 month. Schematic was created using BioRender.com. (TIFF) [file ppat.1011772.s001.tiff]

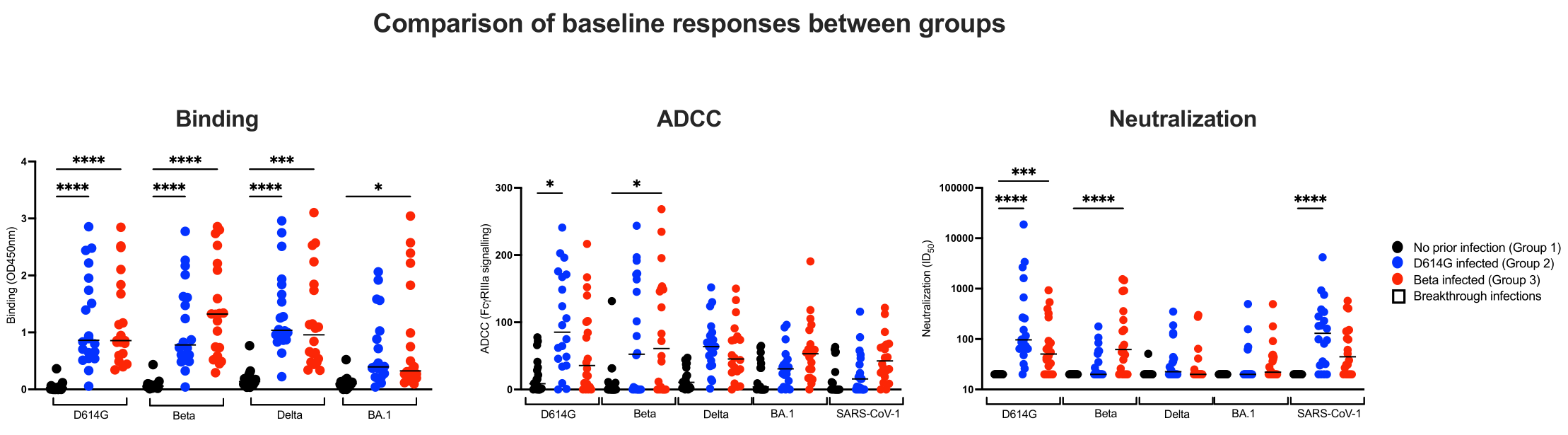

Supplement: S2 Fig — Plasma was tested at baseline (pre-vax) for antibody binding (A), ADCC activity (B) and neutralization assays (C) against D614G, Beta, Delta, Omicron (BA.1) and SARS-CoV-1 for participants with no prior infection (black), participants infected with D614G (blue) or Beta infected participants (red). Responses were compared between the three groups. Binding antibodies were quantified by OD450nm values, ADCC activity was measured in FcγRIIIa signaling and neutralization was indicated by ID50 values. Bars for binding and ADCC represent geometric mean values and bars for neutralization represent geometric mean titer. Statistical significance between the different groups was determined using the Kruskal-Wallis test with Dunn’s correction for multiple comparisons. Significance is shown as: ****p<0.0001, ***<0.001, **p<0.01, *p<0.05. (TIFF) [file ppat.1011772.s002.tiff]

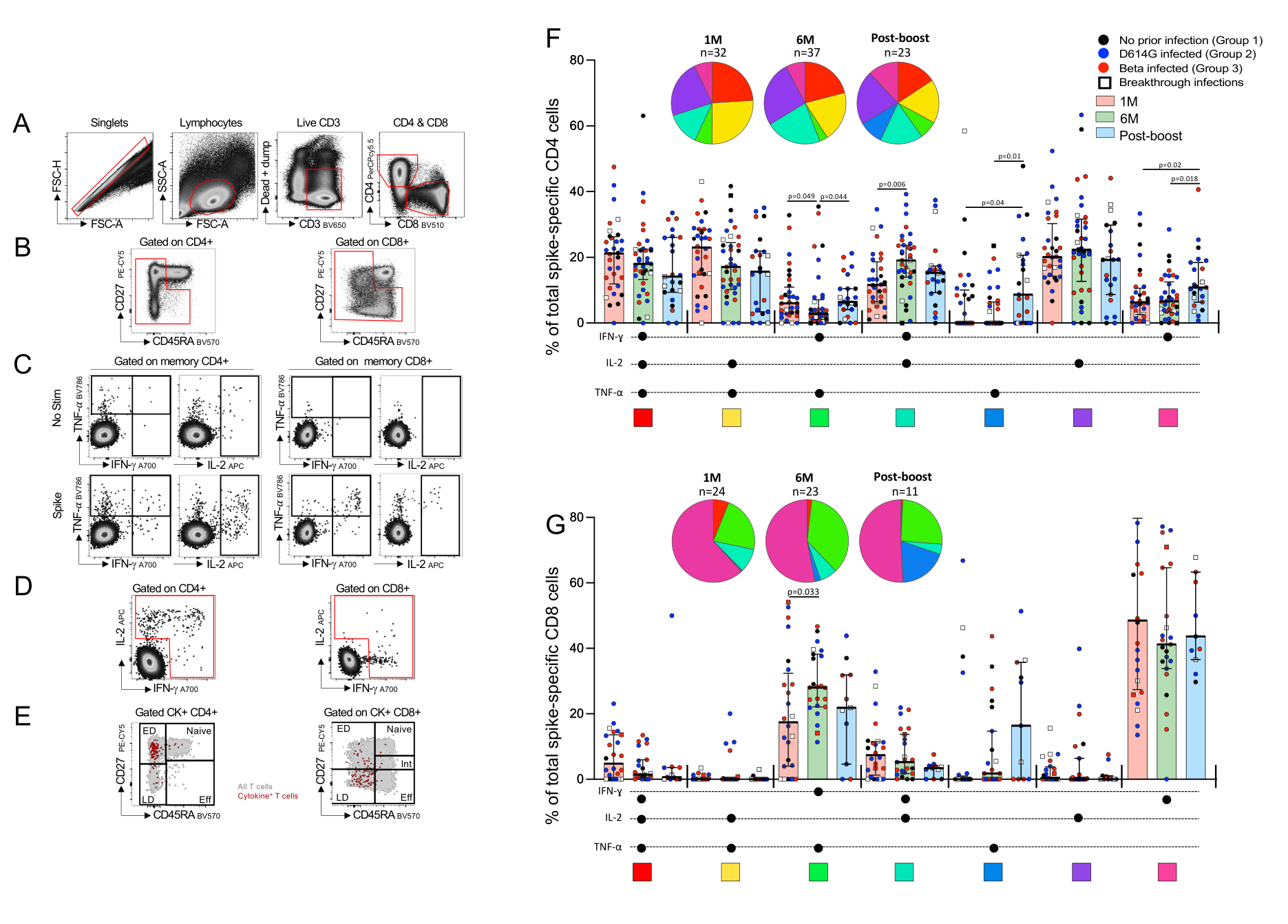

Supplement: S3 Fig — Gating strategy (A and B) and representative examples of SARS-CoV-2 spike-specific IFN-γ, IL-2 and TNF-α production in memory CD4+ and CD8+ T cells (C). (D) Representative examples of SARS-CoV-2 spike-specific IFN-γ or IL-2 production in CD4+ and CD8+ T cells on which memory phenotyping were gated. (E) Representative plots showing spike-specific T cell immune phenotyping. ED: early differentiated, LD: late differentiated, Eff: effector, Inter: intermediate. Polyfunctional profile of SARS-CoV-2-specific CD4+ (F) and CD8+ T cells (G) at the 1M (red bar), 6M (green bar) and post boost (blue bar) time-points. PBMCs from patients with either no prior infection (black), infected with D614G (blue), or infected with Beta (red) are represented by closed circles and BTIs are represented as white squares. The x-axis illustrates each combination which is indicated with a black circle for the presence of IFN-g, IL-2 and TNF-a. The medians and interquartile range are shown. Each response pattern (any possible combination of IFN-g, IL-2 and TNF-a production) is color coded and summarized in the pie charts, with each pie slice representing the median contribution of each combination to the total SARS-CoV-2 responses. The permutation test was used to compare the statistical differences between the pie charts and the Mann Whitney Sum Test to compare response patterns between the time-points; p values <0.05 were considered statistically significant and are bolded. (TIFF) [file ppat.1011772.s003.tiff]

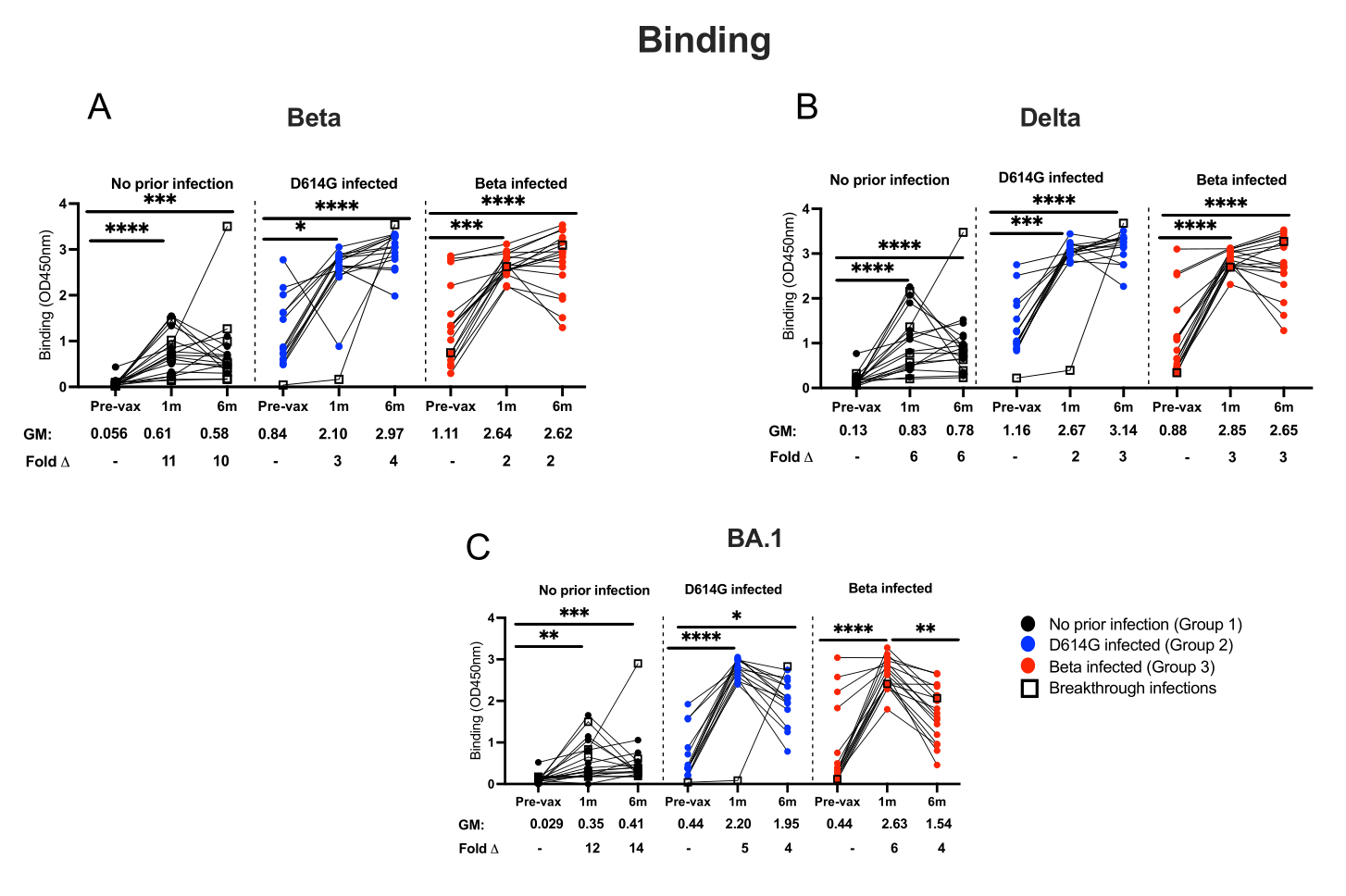

Supplement: S4 Fig — Plasma samples from participants with no prior infection (black), infected with D614G (blue) or infected with Beta (red) were tested for binding responses to the Beta (A), Delta (B) and BA.1 (C) variants at three time points (pre-vaccination, 1 and 6 months post-vaccination). Geometric mean values and median fold changes are shown below the graphs. Fold changes were all calculated relative to the pre-vaccination time points. Binding antibodies were quantified by OD450nm values. All experiments were performed in duplicate. The Friedman test with Dunn’s correction for multiple comparisons was used to determine statistical significance. Significance is shown as: ****p<0.0001, ***<0.001, **p<0.01, *p<0.05. (TIFF) [file ppat.1011772.s004.tiff]

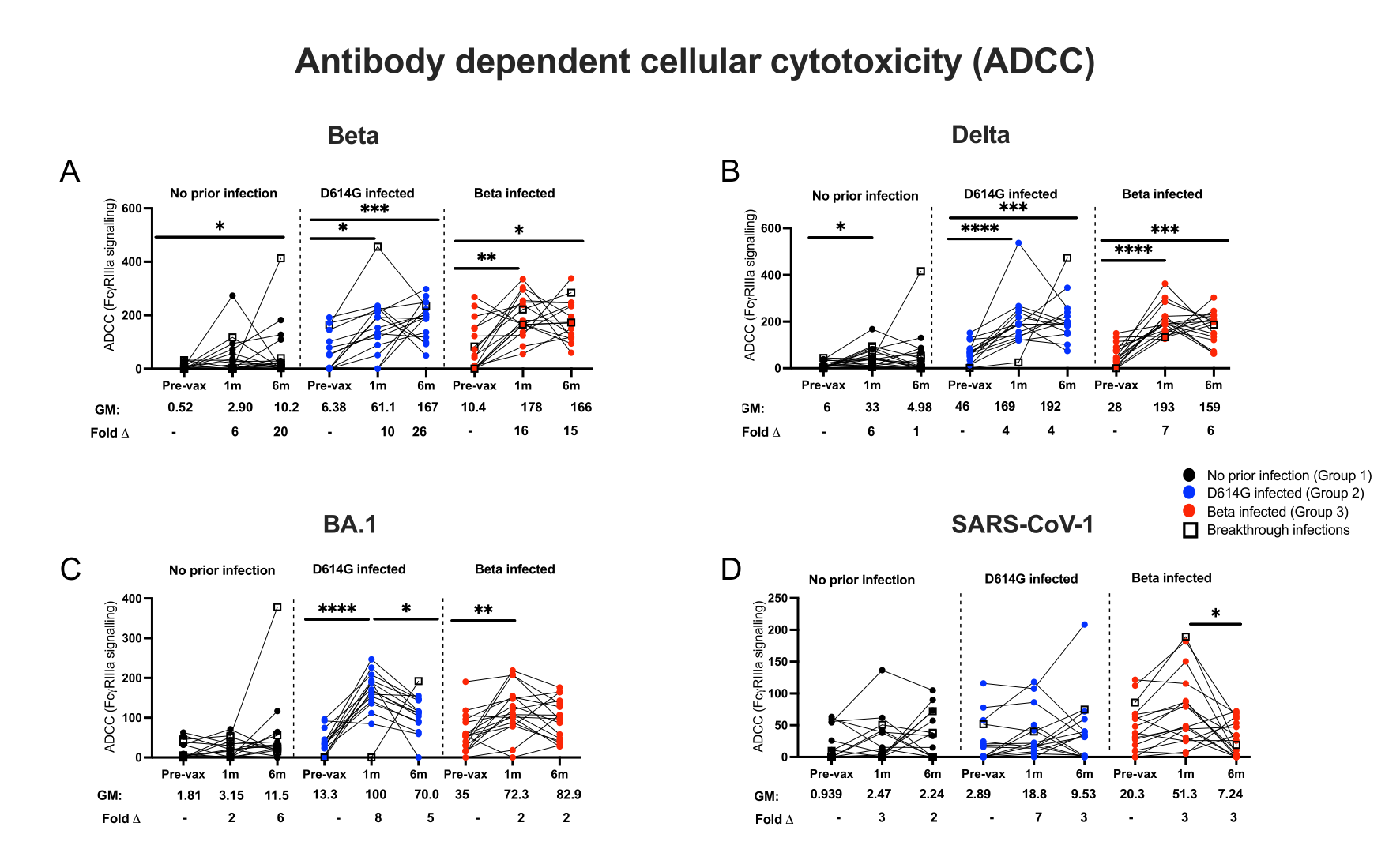

Supplement: S5 Fig — Plasma samples from participants with no prior infection (black), infected with D614G (blue) or infected with Beta (red) were tested for ADCC activity to the Beta (A), Delta (B), BA.1 (C) variants and SARS-CoV-1 (D) at three time points (i.e, pre-vaccination, 1 and 6 months post-vaccination). Geometric mean values and median fold changes are shown below the graphs. Fold changes were all calculated relative to the pre-vaccination timepoints. ADCC activity was measured in FcγRIIIa signaling. All experiments were performed in duplicate. The Friedman test with Dunn’s correction for multiple comparisons was used to determine statistical significance. Significance is shown as: ****p<0.0001, ***<0.001, **p<0.01, *p<0.05. (TIFF) [file ppat.1011772.s005.tiff]

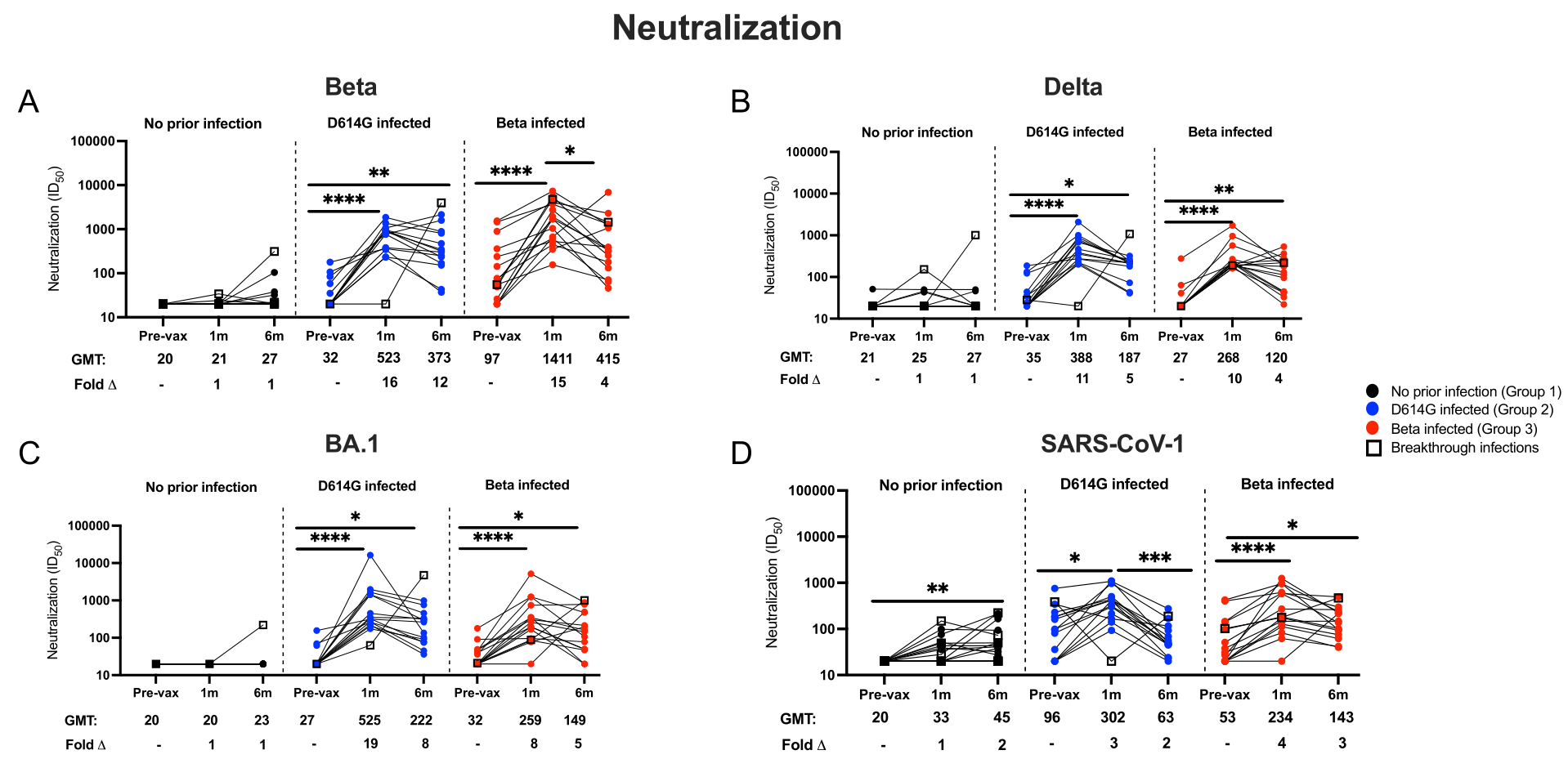

Supplement: S6 Fig — Plasma samples from participants with either no prior infection (black), infected with D614G (blue), and infected with Beta (red) were tested for neutralizing responses to the Beta (A), Delta (B), BA.1 (C) variants and SARS-CoV-1 (D). Plasma was tested over three time points; pre-vaccination, 1 and 6 months post-vaccination. Geometric mean values and fold changes are shown below the graphs. Fold changes were all calculated relative to the pre-vaccination timepoints. Neutralization was indicated by ID50 values. All experiments were performed in duplicate. The Friedman test with Dunn’s correction for multiple comparisons was used to determine statistical significance. Significance is shown as: ****p<0.0001, ***<0.001, **p<0.01, *p<0.05. (TIFF) [file ppat.1011772.s006.tiff]
